# Entrepreneurial Potential and Gender Effects: The Role of Personality Traits in University Students’ Entrepreneurial Intentions

**DOI:** 10.3389/fpsyg.2019.02700

**Published:** 2019-12-04

**Authors:** Alexander Ward, Brizeida R. Hernández-Sánchez, Jose C. Sánchez-García

**Affiliations:** Department of Social Psychology, University of Salamanca, Salamanca, Spain

**Keywords:** entrepreneurship, potential, Spain, university, gender

## Abstract

The percentage of female entrepreneurs is far below the level of males, although it has increased over the past several years. Based on the theory of planned behavior, the purpose of this article is to specify a model in which the relationship among entrepreneurial potential, gender and entrepreneurial intention are explored, by analyzing how perceived behavioral control (PBC) and perceived entrepreneurial skills, as exogenous variables, affect expression of intention for business, and how these are mediated by their entrepreneurial motivations and risk taking propensity. Control variables where also included in this model, such as necessity-driven motives for business, in order to observe whether these are an influential factor. An implementation of Structural Equation Modeling (SEM) was used to analyze data collected from 677 students. Variables within the model were compared by gender using t-Test, and all multivariate analysis were done by each one separately as well in order to better gauge their perceptions. Results showed that mean differences between males and females are not abundant, and come only from intentions, PBC and subjective norm, which are higher in males; and motives for business higher in females. Multivariate analysis shows gender differences at the mediation level and that necessity-driven motives are an influencing factor, more so in males, and it hampers the significance of subjective norm. Finally, the theoretical and practical implications of the results within the framework of entrepreneurship in Spain and future alternatives to improve the entrepreneurial potential are discussed.

## Introduction

Spain was hit by an economic recession in 2008, which affected the country on many levels, including the organizational sector. This lowered entrepreneurship activity rate, and, although has been progressively improving in latest years, it is still among the lowest among the European Union (Peña et al., 2019). Universities, being a brewing spot for knowledge spillover (Audretsch and Caiazza, 2016), are considered as an engine to improve economic growth by developing potential entrepreneurs (Lackéus, 2015), which can help Spain reduce unemployment levels by increasing entrepreneurial activity.

Because of this, educational institutions are generally looked upon with interest, nevertheless, university itself is also a complex equation. First, because of the variability between education fields and occupational interests, and second, because of group differences within them. This study focuses in the second subset, specifically, gender. Literature generally indicates entrepreneurship is a male-dominated field (Muntean and Ozkazanc-Pan, 2015), meaning gender is a highly confounding variable that moderates entrepreneurship behavior and intentions (Haus et al., 2013; Guzman and Kacperczyk, 2019), therefore, should always be accounted for. Even if universities are a brewing spot for entrepreneurs, we argue it must also be understood the variability of the entrepreneurial potential within them by gender (Dilli and Westerhuis, 2018).

Recent reports in Spain that measure the entrepreneurial mindset in the university population, like the Entrepreneurship Observatory (Guerrero et al., 2016) or the GUESS reports, are mostly descriptive and do not fully encompass entrepreneurial potential from a theoretical standpoint (Santos, 2008; Santos et al., 2013), gauge a causal relationship between perceptions, nor study gender differences thoroughly.

The present study responds to this need, and aims to analyze the entrepreneurial potential in a Spanish university context and, specifically, we reach to map how this factor gets affected when accounting to gender. This will be done by mapping the causal relation of personality traits to entrepreneurship intention, on both, males and females, and discuss how it relates to the entrepreneurial development situation in Spain. Entrepreneurial potential is a useful concept because not only it encompasses the degree in which an individual possesses entrepreneurial-related qualities, but also accounts for entrepreneurial intentions, or the state of mind of determination to act toward creating business (Santos, 2008; Santos et al., 2013). Intentions are particularly meaningful because they have a reasonably high prediction power of actual behavior (Krueger, 2017), and it is a good proxy to overview the short-term future of business activity.

Also, although commonly argued and studied, necessity-related variables are seldom included in intention models (Arrighetti et al., 2016). This is a huge gap in literature, as it is an influential variable for business (Lindsay, 2014), and a motive that plays a role for business creation in many countries (GEM, 2019), more so in females (Kelley et al., 2017). Heeding to these two points, this study will (1) Create a causal model for entrepreneurship intentions suited for the Spanish and university context, and analyze it by gender, and (2) Explore how necessity plays a role in their intention and motives for business in its university students and by the inclusion of these into a causal model to understand how future business-oriented initiatives are explained by the current situation in the country (OECD, 2019). Lastly, this study will analyze the models separately by gender, meaning the results for male and females will be presented separately and will allow to better visualize difference and similarities, meaning it goes beyond explaining gender as an influential variable, but also as models.

### Theoretical Background and Conceptual Model

#### The Importance of Gender as a Variable in Entrepreneurship Research

A good way to start this section is by stating that entrepreneurship is not founded on a static profile of people and interests (Kerr et al., 2018; Mwatsika et al., 2018), but it is rather a term attributed to the set of actions that lead to the creation of new products and services, which can vary by, both, interests and opportunity evaluation marked at the individual (Kerr et al., 2018) and group level (Parker, 2018). The latter is an important factor, as literature shows certain differences in the set of business founded by groups, such as gender. In fact, there is strong empirical evidence that male and female entrepreneurship is different in both, business structure (Kelley et al., 2017) and individual goals and thought processes (Minniti, 2009).

Because of this, clear patterns between male and female entrepreneurship can be observed, such as concentration in different types of venture (e.g., males more focused in STEM fields, females in services of highly routine tasks (McCracken et al., 2015; Kelley et al., 2017) and different levels of growth orientation in their business (Klapper and Parker, 2011); as well different goals, action patterns and perceptions about business (Minniti, 2009; Dilli and Westerhuis, 2018). This asymmetry in the entrepreneurial mindset by gender is important because, first, both contribute differently in societal development, as well different sectors and services (OECD, 2016; Hyams-Ssekasi et al., 2019). Second, because said asymmetrical behavior in business is also attributed to gender-held stereotypes, where it generally still holds a male-like vision of business, meaning it is sometimes skewed toward favoring masculine models of behavior (e.g., high profit, high ambition and growth goals (Klapper and Parker, 2011; Balachandra et al., 2017). This has led to argue, and supported by hard evidence, that this factor also impacts differently in how entrepreneurship is perceived by each gender, meaning that the set of factors that influence their mindset are also important to understand and differentiate.

The process of business creation, and indeed, the preceding factors that lead to it, is a different experience for males and females, including perceptual and cognitive factors that lead to develop intentions to start the venturing process (Gupta et al., 2017; Hyams-Ssekasi et al., 2019). This has gained traction in the research field because entrepreneurial potential is also dependent on whether these variables are accounting for gender differences, and in which way they can be factored to increase this potential, but what is this potential in this case?

Potential is a term commonly used in the lexicon, including academia (Gardner and Davies, 2013), but the concept of entrepreneurial potential in a broader, but more theoretical perspective, has been proposed only recently by Santos (2008) and Santos et al. (2013) which are two independent studies which aimed to create a model for this concept. Entrepreneurship potential, according to these two studies, is a set of psychological perceptions and cognitions of oneself related to success, confidence and risk; entrepreneurial competencies, and entrepreneurial motivations, all which impact intentions (Santos et al., 2013). Essentially, entrepreneurial personality traits and environmental variables affecting one’s intention for business can be said that comprises what is called entrepreneurial potential, which is the set of variables we are going to factor in this study to analyze gender differences.

#### What Makes Males and Females Differ in Their Development of Their Entrepreneurial Potential and Intentions?

##### The role of cognitive and skill factors

According to our theoretical research, the evidence strongly points that an asymmetrical behavior in entrepreneurship between genders roots down to the following reasons. First, that entrepreneurship as a career choice has been argued that currently adjusts better with male, rather than female, traits, especially, attitudinal, behavioral and motivationally (Muntean and Ozkazanc-Pan, 2015). Some theories, like the person-fit theory (Caplan, 1987) and the attraction-selection-attrition model (Schneider, 1987) suggest people would self-select occupations more akin to their personality, competences and values. If any of this holds true, we argue it would lead to a higher proclivity in males to pursuit entrepreneurship, as it could work as a pull factor. According to the latest report on student’s attitude toward entrepreneurship in Spain (Guerrero et al., 2016), a higher percentage of males expressed intentions or some determination toward being entrepreneurs. This result is not uncommon (e.g., Wilson et al., 2004, 2007; Zhao et al., 2005; Gupta et al., 2008) so it would be reasonable to expect this pattern in our study.

Some other attitudinal traits, like how people perceive controllability and efficacy over certain actions, has shown to be a substantial predictor of intentions in entrepreneurship (Zhao et al., 2005; Krueger, 2017). Males generally score higher in these perceptions for entrepreneurship (Koellinger et al., 2008), although, it’s causal relation to intentions have been found to be non-significantly different between genders (Zhao et al., 2005). Given this, we expect to find difference in their perceptions by gender in their mean, but non-different causal predictions to intention in both. Instead of using two separate variables (efficacy and controllability), we use Perceived Behavioral Control (PBC) on entrepreneurship, as its better suited to our context and allows us to explore perceived entrepreneurial behavior. Also, due to the hierarchical nature of this factor (Ajzen, 2002), we can explore both, perceived controllability and efficacy. For the purposes of this study, PBC is defined as “people’s expectations regarding the degree to which they are capable of performing a given behavior, the extent to which they have the requisite resources and believe they can overcome whatever obstacles they may encounter” (Ajzen, 2002, p. 676).

*Hypothesis 1*: For both genders: perceived behavioral control of possible entrepreneurial activities has a significant positive effect on entrepreneurial intentions *not* significantly different from each other.

Part of these resources, which can be internal or external, are also skills and knowledge (Bandura, 1997). There is an assumption that certain things “entrepreneurial” can be acquired through training and exposure (Kuratko, 2005), which suggests entrepreneurial behavior is a rather complex interaction between predispositions (Zhang et al., 2009; Zhao et al., 2010), perceptions (Arenius and Minniti, 2005), and skills, the latter which can be honed (Lackéus, 2015). In other words, people can, to some extent, become entrepreneurial. The evidence of education on entrepreneurial intentions is somewhat shady, but there is evidence of certain gains from it, according to two recent meta-analytic studies (Martin et al., 2013; Bae et al., 2014). Some authors have also found that males and females who would perceive themselves as entrepreneurially-competent would also be more likely to set up business, however, it becomes specifically important for the latter (Arenius and Minniti, 2005; Minniti and Nardone, 2006; Langowitz and Minniti, 2007). Additionally, Sánchez (2013) and Zhao et al. (2005), among others, found that education positively impacts self-efficacy. We hypothesize that perceived skills and PBC work together to enhance a feeling of capability for business, and increase the likelihood of venture creation through intentions. Given our population are related to higher education, we do not expect males and females to perceive their entrepreneurial skills significantly different from each other, as both come from an educational platform that allows exposure to either develop or improve certain skills that would make them feel more entrepreneurially competent. Also, given the special relation of skills as a resource in females, we are specifically interested in measuring its effect separately from PBC.

*Hypothesis 2*: For both genders: perceiving to have entrepreneurial-related skills and competences has a significant, positive effect on entrepreneurial intentions that is *not* significantly different from each other.

##### The role of motivational factors

Competences by itself is likely not enough reason for people wanting to be entrepreneurs. This interest for business could also be due to them personally wanting to, perceiving that an intrinsic reward or personal goals can be achieved through entrepreneurship, making it desirable (Krueger and Brazeal, 1994). There is also certain variability in these motives between gender, like females looking for independence (Sánchez Cañizares and Fuentes García, 2010), males for profit gains (Maes et al., 2014), and both equally look for personal satisfaction (Buttner and Moore, 1997). It is this intrinsic motivation to obtain personal achievements what pushes a certain disposition to act upon that goal (Ryan and Deci, 2000). We believe that motivations mediate personality and behavior, and that this development of motives toward entrepreneurship could get boosted by the fact that people can confidently act on their interest for it. In other words, perceived controllability and efficacy, knowledge, and skills could influence their motives to pursue business, as it signals venture creation as an achievable goal. Given perceived PBC generally shows gender differences in their mean, and skill correlates strongly with female interests for business (Minniti, 2009), we expect to find some variation in how these impact their motives, and hypothesize that skillfulness is what strengthens motives for entrepreneurship in females, rather than perceived control. Conversely PBC will create a higher impact in males.

*Hypothesis 3*: Intrinsic motives for entrepreneurship mediate the positive effect of PBC on intention, which is stronger in males.

*Hypothesis 4*: For both genders: intrinsic motives for entrepreneurship mediate the positive effect of skills on intentions and is *not* significantly different from each other.

These perceptual relations, on the other hand, can be influenced by environmental pressures, which leads to our next point.

##### The role of environmental factors

Entrepreneurship itself creates different environments with different challenges for males and females (Jennings and McDougald, 2007) some of which could relate to stereotypes (Marlow and Patton, 2005) that could create a self-imposed barrier for females (Langowitz and Minniti, 2007). In other words, it becomes harder for them to succeed or are primed to not pursue it, which has been evidenced by studies that have shown a certain bias to disfavor traits more commonly found on females (Balachandra et al., 2017), or stereotyping effects that create a cascade on perception toward the entrepreneur stereotype (e.g., Gupta et al., 2008, 2017). It has also been found that a substantial portion of females are focused in different business sectors, generally of low income or small business, which becomes harder to finance or get support because reputation limitations and lack of interest on investors for these type of ventures (Klapper and Parker, 2011).

We do not have the power within our dataset to explain relationships with variables strictly related to stereotype, or how these are reflected in the perceptions of would-be entrepreneurs, so we will use subjective norm as a way to infer how perceived support could affect intention and motives on a more general level. To this, Baughn et al. (2006) found that positive perception about entrepreneurship by society has a stronger effect on females than on males to pursue entrepreneurial activities, also that gender equality does not raise the number of female entrepreneurs (a finding also shared by Sarfaraz et al., 2014), however, the perception of female entrepreneurs becomes more positive in these societies, which encourages stronger on women for startups. Given how female empowerment and inclusion in the labor market could enhance their entrepreneurial pursuits, we guided ourselves with the Gender Inequality Index from the United Nations Development Programme to posit our next hypothesis. Its latest report (UNDP, 2018) ranks Spain 14th worldwide on gender equality (out of 189 countries included), so it would be reasonable to find a positive effect from social perception with both groups, and that females are being encouraged. Following the study of Baughn et al. (2006), we also hypothesize that this would weight stronger on females on its motives, thus mediating its positive effect on intention.

*Hypothesis 5*: For both genders: positive social perceptions toward entrepreneurship has a significant, positive effect on entrepreneurial intentions that is *not* significantly different from each other.

*Hypothesis 6*: For both genders: intrinsic motives for entrepreneurship mediate the positive effect of social perceptions on entrepreneurship on intentions and it’s significantly *stronger* on females than males.

Lastly, the rate of entrepreneurship activity relates to economic development and affects both genders, however, becomes more impactful in females in a way that fluctuates more drastically, even closing TEA gaps in certain countries with low development due to necessity-driven factors (Kelley et al., 2017). This third variable moderates the gap in a way that will narrow, disappear, or even invert, if women need to be entrepreneurs, but widen if they don’t.

This means that environments high in uncertainty and unemployment may lead people to become riskier by effect due to the necessity to have income, where self-employment becomes a useful source (Hofstede et al., 2004). Proclivity to take risks has also been found to vary by gender in many of its domains, males generally scoring higher (Nicholson et al., 2005), however, the latter found females score higher proclivity on career and social domains. For intention-based theory, Zhao et al. (2010) also found risk propensity has a positive effect on entrepreneurial intentions, meaning it is a useful variable to analyze.

Due to the unemployment situation in Spain (OECD, 2019), we hypothesize that necessity will positively spike, on both, their perception of how risky one must get to achieve goals, and it will not dampen any causal path neither on males or females, due to their competence to bear it. Some authors have studied risk propensity as an endogenous variable, and found it also acts as a mediator to entrepreneurial intentions (Altinay et al., 2012) as well increases with the perception of self-efficacy and confidence (Densberger, 2014). From this, we hypothesize riskiness will also depend on whether the individual has some measurable competence to act, thus mediating its effect on intention. In other words, even if both express high fear of uncertainty for entrepreneurship (Guerrero et al., 2016; Peña et al., 2019), we are expecting risk taking propensity to positively predict and mediate any effect on entrepreneurship intentions. We propose a similar effect from Motives, where in females the interaction may be more skill-dependant.

*Hypothesis 7*: For both genders: risk taking propensity has a significant, positive effect on entrepreneurial intentions that is *not* significantly different from each other.

*Hypothesis 8*: For both genders: risk taking propensity mediates the positive effect between PBC and intentions, and this effect is significantly *stronger* in males.

*Hypothesis 9*: For both genders: risk taking propensity mediates the positive effect between perceived entrepreneurial skills and intentions, and is *not* significantly different from each other.

*Hypotheses 10 and 11*: For both genders: Perceived control (H10), knowledge and skills (H11) on entrepreneurship positively increase their motives to create business, which is partially why they become riskier to pursue it, having a positive effect in its intentions.

**FIGURE 1:**
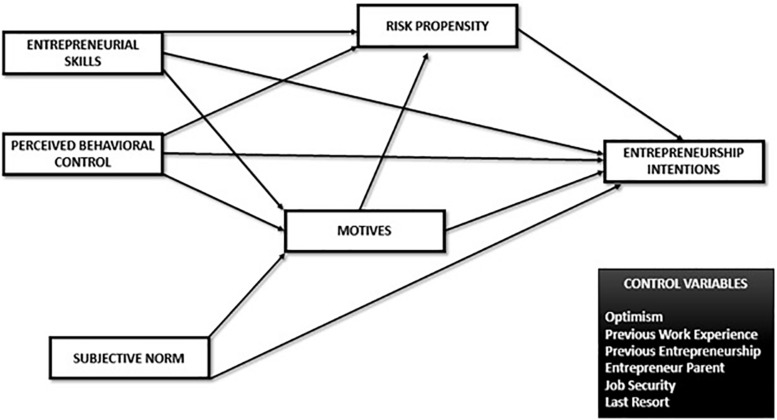
Structural model.

Given these hypothesis, our structural model proposes how these variables interact on entrepreneurial intention in Figure 1:

Due to having some variables at our disposition that could be influential, we control for their effects, as are expected to be confounding to the responses on some of our main variables. Previous work experience has been found to affect interest on entrepreneurship (Arenius and Minniti, 2005), as well entrepreneurship experience in itself (Miralles et al., 2015); and having entrepreneurial parents (Kuckertz and Wagner, 2010), although this one is disputable on intentions (Rachmawan et al., 2015). Some authors have also found optimism to be a driver for venture creation, as it taps into the perception that their projects will have success (e.g., Ozaralli and Rivenburgh, 2016), thus, we include it. Given these two inspire to act, we will control for their effects on risk taking propensity as well.

Lastly, we suspect some of our relationships would change if we control for certain effects from economic-related responses. As the Spanish population is still suffering from high unemployment rates (OECD, 2019), we believe that environmental pressures, mainly due to economic stress or lack of employment would affect entrepreneurial intentions. In fact, some regions have switched from opportunity-driven to necessity-driven (Cruz del Rio Rama et al., 2014). Heeding to this particular interest, we will measure two versions of this model. Model 1 will not include two control variables we show in the model: “Job Security,” “Last Resort.” Model 2 will control for its effects. This will allow to evaluate how some interactions are explained due to their presence.

## Materials and Methods

### Sample and Procedure

#### Sampling

Participants were selected using stratified sampling. This was specifically done as: (1) Autonomous Communities in Spain are bound to have cultural and political variations, and (2) the study was not specifically targeted toward business students, and different academic fields can also show differences in entrepreneurial behavior. In order to increase representativeness of the Spanish and student population, it specifically aimed to sample students considering as much Autonomous Communities and academic fields as possible.

Sampling was done from September 2017 to June 2018, through the collaboration of educational contacts across universities and Autonomous Communities, which prompted their students to take our questionnaire. Before students were given the questionnaire, they were to sign an informed consent, which specified the objective of the study, as well the protection of their data, which included animosity.

After the informed consent was signed, they were given a link to answer the questionnaire, which was taken online through a platform designed specifically for the use of our scale (Sánchez-García, 2016). The same can be found at http://peul.jssm.es/English. Participants were instructed on how to access the questionnaire, and how to answer it. The questionnaire had no time limit, but was estimated to last for approximately 30 min for completion.

#### Participants

The resulting sample comprised of 677 university students from Spain, representing 19 provinces and 12 universities. Age ranges from 15 to 55 years of age, with an age mean of 22. Table 1 breaks down the demographic details from our sample by sex and academic field, and Table 2 by Autonomous Community.

**TABLE 1:** Sample demographics.

**Type**	**Male**		**Female**		**Total**	
	**N**	**%**	**N**	**%**	**N**	**%**
Sex	170	25.1	507	74.9	677	–
Work experience	98	57.6	239	47.4	328	48.4
Entrepreneurship work experience	7	4.1	2	0.4	9	1.3
Social sciences	43	25.3	141	27.8	184	27.2
Humanities/Arts	2	1.2	4	0.8	6	0.9
Architecture	3	1.8	7	1.4	10	1.5
STEM	19	11.2	21	4.1	40	5.9
Education	43	25.3	154	30.4	197	29.1
Business/Management	16	9.4	56	11	72	10.6
Law	4	2.4	30	5.9	34	5
Agriculture	3	1.8	6	1.2	9	1.3
Nurse	0	0	1	0.2	1	1
Didn’t specify	37	21.8	87	17.2	124	18.3
*n* = 677 University Students; Number of provinces = 19; Number of Universities = 12						
Age range = 15–55; Age mean = 22						

**TABLE 2:** Participants by autonomous community.

**Autonomous community**	**No.**
Andalusia	134
Aragon	4
Asturias	37
Cantabria	9
Castilla La Mancha	16
Castile and Leon	165
Catalonia	121
Community of Madrid	65
Valencian Community	11
Extremadura	72
Galicia	6
Balearic Islands	2
Canary Islands	4
La Rioja	8
Navarre	8
Basque Country	8
Region of Murica	6

From our sample, 25% were males, and 75% females. As for the specifics per field, on Social Sciences, we only had samples from Psychology, which means all samples from that category belong to that field. For Humanities, our sample represents fields related to language, history and literature. Architecture was treated as a separate field, as it is harder to fit into either STEM or Humanities/Arts, and did not provided any specialty within it. For STEM fields, our sample comprised of engineering and mathematics. For Business and Management: tourism, economics, management and human resources. For Law: criminologists and international relations. Our sample did not respond to any specialization on Agriculture, so we do not have any clear range. For specifically health-related, we only had one Nurse. Out of our sample, 124 participants, or 18.3%, did not specify their academic field. To this data, more than half of our sample (56.3%) comes from Psychology and Education students, followed by Business (10.6%), and fourth, STEM (5.9%).

### Instrument

The scale applied utilizes a questionnaire format, in which either statements or questions must be answered in interval metrics (Likert scoring from 1 to 5). Specifically, the scale (Sánchez-García, 2016) aims to measure entrepreneurial competences and related attitudes through the following composite variables: PBC (5 items), Entrepreneurial Skills (5 items), Subjective Norm (3 items), Motives (4 items), Risk Taking Propensity (4 items), and Entrepreneurship Intention (6 items). These are detailed below:

Perceived Behavioral Control (PBC; α = 0.881, ω = 0.884) frames the level of agreement in which an individual believes it has the ability to control actions related to venture creation, as well the confidence of its performance over it. We include a mixture of perceived controllability and perceived efficacy in its item structure, as they share high commonality and work as a second-order hierarchy (Ajzen, 2002). An item example of controllability is: “I can control the processes of creating a new company,” and an example of self-efficacy is “Starting a business would be easy for me.”

Entrepreneurial Skills (Skill; α = 0.837, ω = 0.834) frames the level on which an individual possess a specific set of skills associated with entrepreneurs. This is answered by self-assessing each skill, and asked in accordance to an entrepreneurial framework. An example would be to frame the degree of opportunity detection skills, ranging from none, to high.

Subjective Norm (SubNorm; α = 0.738, ω = 0.781) frames the level of agreement in which an individual perceives that entrepreneurial activities are favored or sponsored within different social circles. An item example is: “My closest friends value entrepreneurial activity above other activities.”

Personal Motives (Motives; α = 0.764, ω = 0.767) frames the level of agreement in which an individual would use entrepreneurial activities to pursue specific personal objectives. An example among these items would be to start a business “For a feeling of personal fulfillment”.

Risk taking propensity (RiskProp; α = 0.738, ω = 0.745) frames the level of agreement in which a person is willing to take risks to obtain desired outcomes. An item example is: “I think it’s worth taking risks to obtain better rewards.” This variable does not measure entrepreneurial-specific risks, but the general measure of a person to take them.

Entrepreneurship Intention (Intention; α = 0.939, ω = 0.936) frames the level of agreement in which a person is determined to start a business. An item example is: “I have the firm intention to start a business 1 day.”

The six control variables for our model are the following:

Optimism (Optim; α = 0.839, ω = 0.847) frames the level of agreement in which a person believes that their future holds positive outcomes, or that there is a positive side of every experience. An item example is: “No matter how bad things can go, I always find something positive.”

Three dichotomous variables were used, which indicate whether the individual has had previous experiences, both as an employee (WorkExp) and/or self-employed (EntWorkExp), and if they have at least one parent that is self-employed (EntParent). These are answered exclusively through “no”-0 or “yes”-1.

Lastly, two items (Likert scoring, 1–5) measure necessity-based motives to pursue entrepreneurship. Entrepreneurship for a Secured Job (JobSec) frames the level of agreement in which an individual would pursue entrepreneurship because it would bring them the security of being employed. Entrepreneurship as Last Resort (LastRes) frames the level of agreement in which an individual would pursue entrepreneurship due to the fact that there is no other job alternative.

### Statistical Procedure

Given the multivariate nature of the model, and our interest to measure causal relations between these, we used Structural Equation Modeling (SEM) to achieve this objective. The software AMOS 23 was used for our analysis, as it has become a well-known structural equation software for multivariate analysis (Nachtigall et al., 2003). For correlations, descriptive and mean comparison, we used SPSS 23.

#### Model Fit

Conventionally, the method for evaluating fit in a model is the Chi square (χ^2^) statistic, which should not be significant in the case of a good fit, however, this index can be ignored if the sample size obtained for the study is greater than 200 (Jöreskog and Sörbom, 1996), given its sensibility to sample size. As it is the case, other indices were considered for model fit. These included the Comparative Fit Index (CFI), the Tucker-Lewis Index (TLI), the Adjusted Goodness of Fit (GFI), the Root-Mean-Square Error of Approximation (RMSEA), and Expected Cross Validation Index (ECVI). Squared multiple correlations (R^2^) were also taken into account, as it shows how much of the variance of the independent variables are explained by the predictors in the model.

#### Direct, Indirect, and Moderation Effects

To estimate the coefficient and significance of direct effects, we used Maximum Likelihood Estimate. To analyze mediation effects and group differences, Bootstrap was used with 2000 iterations and 0.95 bias-correction. We tested whether the effect between variables are statistically significant by taking the product or difference between the unstandardized regression weights on the mediation or moderation path of interest: for mediation (A×B), or (A×B×C), and for moderation (A–B) to generate standard errors and confidence intervals, then from those calculate a p-value at the 95% confidence level, as this shows sufficient to demonstrate adequate mediation and moderation effects (Hair et al., 2010). The alpha value of *p* < 0.05 for statistical significance was used in all our analyses.

#### Mean Comparison Between Genders

Lastly, to compare the mean difference between males and females with the variables within the model, the t-Test statistic was used. Homogeneity assumptions were considered for results interpretations. Accordingly, Levene’s test was used to observe whether there was homogeneity within each variable. Variables who yielded statistically significant results (<0.05), were analyzed under the assumption that are not homogeneous. The software SPSS 23 includes results of the t-test from both assumptions (homogeneity and non-homogeneous), thus, we selected the results in accordance to the significance level of Levene’s Test.

## Results

### Model Fit

The model and the hypotheses interactions hold the criteria for analysis when considering the correlation values obtained which are shown in Table 3. Model fit indices for SEM resulted in: CFI = 0.992 Model 1, 0.990 Model 2; TLI = 0.924 Model 1, 0.903 Model 2; GFI = 0.994 Model 1, 0.990 Model 2; RSMEA = 0.033 in both models; and ECVI = 0.457 Model 1, 0.435 Model 2. Fit indices thresholds used are from three different sources: (1) from Hair et al. (2010), CFI > 0.90; GFI > 0.95; and RSMEA < 0.05. (2) Threshold for TLI used is from Awang (2012), which is >0. 90, and (3) the ECVI from Browne and Cudeck (1992), while it has no particular threshold indices, it is assumed that the smaller the index, the better the fit and the better the model can predict future sample covariance. According to these thresholds, both models show good fit, and above commonly accepted standards, meaning the proposed models account for the correlations between the variables found in the dataset.

**TABLE 3:** Correlations.

	**1**	**2**	**3**	**4**	**5**	**6**	**7**	**8**	**9**	**10**	**11**	**12**
1-SubNorm	1											
2-PerCnt	0.270^∗∗^	1										
3-Skill	0.180^∗∗^	0.383^∗∗^	1									
4-RiskProp	0.056	0.191^∗∗^	0.273^∗∗^	1								
5-Motives	0.129^∗∗^	0.230^∗∗^	0.277^∗∗^	0.254^∗∗^	1							
6-Optim	0.027	0.263^∗∗^	0.320^∗∗^	0.267^∗∗^	0.196^∗∗^	1						
7-WorkExp	0.004	0.136^∗∗^	0.080^∗^	0.092^∗^	0.064	0.122^∗∗^	1					
8-EntWorkExp	0.044	0.161^∗∗^	0.077^∗^	0.024	0.072	0.022	0.120^∗∗^	1				
9-EntParent	0.110^∗∗^	0.022	0.010	0.053	–0.028	0.019	–0.111^∗∗^	–0.053	1			
10-JobSec	0.191^∗∗^	0.115^∗∗^	0.093^∗^	0.017	0.0275^∗∗^	–0.040	–0.066	0.002	0.060	1		
11-LastRes	–0.014	–0.157^∗∗^	–0.127^∗∗^	–0.117^∗∗^	–0.136^∗∗^	–0.127^∗∗^	–0.040	0.012	–0.050	0.029	1	
12-Intention	0.366^∗∗^	0.627^∗∗^	0.0395^∗∗^	0.222^∗∗^	0.296^∗∗^	0.174^∗∗^	0.062	0.117^∗∗^	0.061	0.236^∗∗^	–0.218^∗∗^	1

**^∗∗^Correlation is significant at the 0.01 level (2-tailed); ^∗^Correlation is significant at the 0.05 level (2-tailed), Pearson Correlation*.*

R^2^ values are also adequate, explaining in males 52 and 56% of the variance of the dependent variable in Model 1 and Model 2, respectively, and in females 44 and 46%. Correlations between the variables, which can be found in Table 3, also follow the expected patterns which correspond to theoretical literature (e.g., a strong and significant correlation of PBC with Intention), and with the proposed path model (i.e., expected significant relations yield significant correlation levels). Results overall suggest the theoretical model and the dataset show the theoretical coherence expected for analysis. Given these results, we proceed with testing our hypotheses.

### Regression Weights

Before proceeding with the results from the path model, we show each individual regression within it in order to further understand how each variable interacts individually, including control variables. Regressions for males are presented in Table 4, and for females in Table 5.

**TABLE 4:** Regression weights: males.

	**Model 1**			**Model 2**		
	***B***	**S.E.**	***p***	***B***	**S.E.**	***p***
Motives ← Skill	0.237	0.076	0.002^∗∗^	0.190	0.072	0.008^∗∗^
Motives ← EntWorkExp	0.146	0.252	0.562	0.169	0.236	0.473
Motives ← WorkExp	0.047	0.101	0.641	0.048	0.095	0.616
Motives ← PBC	0.207	0.066	0.002^∗∗^	0.172	0.062	0.006^∗∗^
Motives ← SubNorm	–0.011	0.068	0.870	–0.047	0.065	0.473
Motives ← LastRes	–	–	–	–0.087	0.037	0.017^∗^
Motives ← JobSec	–	–	–	0.175	0.041	^∗∗∗^
RiskProp ← Motives	0.165	0.076	0.029^∗^	0.165	0.076	0.029^∗^
RiskProp ← PBC	0.016	0.065	0.805	0.016	0.065	0.805
RiskProp ← Skill	0.111	0.079	0.162	0.111	0.079	0.162
RiskProp ← Optim	0.260	0.087	0.003^∗∗^	0.260	0.087	0.003^∗^
RiskProp ← EntWorkExp	0.031	0.247	0.902	0.031	0.247	0.902
Intention ← PBC	0.626	0.075	^∗∗∗^	0.612	0.071	^∗∗∗^
Intention ← Skill	0.349	0.092	^∗∗∗^	0.324	0.087	^∗∗∗^
Intention ← WorkExp	–0.144	0.114	0.204	–0.139	0.108	0.198
Intention ← EntWorkExp	0.052	0.28	0.853	0.097	0.265	0.714
Intention ← RiskProp	0.054	0.086	0.530	0.057	0.082	0.487
Intention ← SubNorm	0.175	0.076	0.021^∗^	0.141	0.073	0.053
Intention ← Motives	0.140	0.086	0.105	0.009	0.087	0.914
Intention ← Optim	–0.118	0.1	0.240	–0.123	0.097	0.203
Intention ← EntParent	0.080	0.118	0.495	0.071	0.112	0.522
Intention ← LastRes	–	–	–	–0.117	0.042	0.006^∗∗^
Intention ← JobSec	–	–	–	0.178	0.048	^∗∗∗^

**Model 1 without Necessity and Model 2 with Necessity. B- Unstandardized estimates; ^∗∗∗^p = 0.001 or less; is significant at the < 0.05 value, ^∗∗^*p* < 0.01; ^∗^*p* < 0.05, Maximum Likelihood Estimation.**

**TABLE 5:** Regression weights: females.

	**Model 1**			**Model 2**		
	***B***	**S.E.**	***p***	***B***	**S.E.**	***p***
Motives ← Skill	0.171	0.037	^∗∗∗^	0.162	0.036	^∗∗∗^
Motives ← EntWorkExp	0.386	0.418	0.355	0.465	0.406	0.252
Motives ← WorkExp	0.044	0.053	0.405	0.068	0.051	0.186
Motives ← PBC	0.063	0.036	0.079	0.046	0.035	0.192
Motives ← SubNorm	0.077	0.034	0.024^∗^	0.049	0.033	0.144
Motives ← LastRes	–	–	–	–0.032	0.021	0.119
Motives ← JobSec	–	–	–	0.118	0.022	^∗∗∗^
RiskProp ← Motives	0.194	0.049	^∗∗∗^	0.194	0.049	^∗∗∗^
RiskProp ← PBC	0.054	0.040	0.175	0.054	0.040	0.175
RiskProp ← Skill	0.150	0.044	^∗∗∗^	0.150	0.044	^∗∗∗^
RiskProp ← Optim	0.158	0.045	^∗∗∗^	0.158	0.045	^∗∗∗^
RiskProp ← EntWorkExp	–0.330	0.466	0.479	–0.330	0.466	0.479
Intention ← PBC	0.577	0.044	^∗∗∗^	0.551	0.044	^∗∗∗^
Intention ← Skill	0.136	0.048	0.004^∗∗^	0.131	0.047	0.005
Intention ← WorkExp	–0.038	0.064	0.556	–0.031	0.063	0.626
Intention ← EntWorkExp	–0.182	0.505	0.718	–0.052	0.497	0.916
Intention ← RiskProp	0.098	0.048	0.041^∗^	0.098	0.047	0.038
Intention ← SubNorm	0.229	0.042	^∗∗∗^	0.220	0.041	^∗∗∗^
Intention ← Motives	0.211	0.054	^∗∗∗^	0.167	0.055	0.002
Intention ← Optim	–0.061	0.050	0.225	–0.053	0.049	0.284
Intention ← EntParent	0.063	0.068	0.355	0.037	0.067	0.576
Intention ← LastRes	–	–	–	–0.089	0.025	^∗∗∗^
Intention ← JobSec	–	–	–	0.073	0.028	0.009

**Model 1 without Necessity and Model 2 with Necessity. B- Unstandardized estimates; ^∗∗∗^p = 0.001 or less; is significant at the < 0.05 value, ^∗∗^*p* < 0.01; ^∗^*p* < 0.05, Maximum Likelihood Estimation.**

Regarding the three exogenous variables, all three significantly predict entrepreneurship intention, except SubNorm for males in Model 2. Out of these three variables PBC has the strongest regression in intentions. This coefficient difference is significant from the other exogenous variables on each group: for males, *p* = 0.015 when compared to Skill, *p* = 0.001 to SubNorm. For females, *p* = 0.001 to Skill, *p* = 0.001 to SubNorm, in both models. Differences between groups are not statistically significant (*p* = 0.424 for PBC, 0.131 for Skill, and 0.414 for SubNorm), however, males show higher coefficients on PBC and Skill, while females on SubNorm. Males and females perceptions on these variables affects intentions not significantly different from each other, however, although statistically it cannot be said that there is a significant difference between groups, separately, SubNorm is not a significant predictor of Intention in Model 2 for males, while it is on females.

PBC and Skill has a statistically significant regression to Motives in males in both models, however, neither impact significantly RiskProp. For females, PBC has no significant regression in RiskProp nor Motives, but Skill has on both. SubNorm has no significant impact in males for Motives in neither model, and in females this regression is significant only in the first model, hinting necessity-related factors may play a role in the impact this factor has.

RiskProp significantly impacts entrepreneurial intentions in females, but not in males, at least when considering the variables within our structural model. We ran a univariate regression analysis on RiskProp to Intention on males, and yielded significant results (*p* = 0.000, *B* = 0.224), which suggests that in a more complex model, risk propensity simply becomes an non-significant, smaller effect, in comparison to other variables, and thus, a more accurate measure of its role in males.

In the second model, Motives’ impact on intentions get affected the most, although this effect is drastically stronger on males. For this group, Motives’ regression on intentions plummets to less than 0.01, and SubNorm has a negative (non-significant) effect on Motives. We tried to determine which of the necessity variables was drowning off the Motives effect to intentions by analyzing the model without each one, and found JobSec would substantially lower the effect, from *B* = 139, *p* = 0.109 to *B* = 0.041, *p* = 0.641. LastRes would lower the effect to *B* = 0.114, *p* = 0.185. This is due to having direct paths of these variables to Intention as well, and it also means that JobSec may have a stronger role on intentions than Motives in males. We also explored whether this was due multicollinearity, but it was not the case [Variance Inflation Factor (VIF) is less than 2 for all variables].

LasRes shows to have a significant negative impact on Motives for males, but on females was not significant, and a significant negative impact in intentions for both. JobSec on the other hand, also impacts significantly, but positively, on Motives and Intentions on both genders, which suggests certain aspects of necessity, at the least, drive entrepreneurial interests in an environment where economic stress pressures on occupational choices.

Of the other control variables, Optim, affects (albeit not within our accepted range for significance) negatively on Intentions, but positive and significant on RiskProp in both genders. WorkExp and EntWorkExp have non-significant, high *p*-value, negative effects on most interactions, except in Motives (not-significant, but positive). Both, Optim and WorkExp, impact negatively in Intentions, and, even if the effect is not significant, the direction itself was not expected. Our only reference to contrast was a study that found optimism was not significant for intentions in Spanish students, but the effect was positive (Giacomin et al., 2016), as such, ours contradicts it. Having at least one entrepreneur parent also has no statistically significant effects on Intentions for neither gender in any of the two models. This was half-expected, as other authors have also found this (Rachmawan et al., 2015). The number of participants who had previous entrepreneurial experience is so small (seven males, two females) that it is hard to make any sense out of the results, but its coefficient to intentions is negative as well. If anything can be inferred from these control variables, is that entrepreneurship is not well perceived. Even if most were not significant, almost all directions pointed negative.

### Path Model Effects

Tables 6, 7 shows the results obtained from our path model by males and females, respectively. Model 2 presents how the inclusion of necessity-related responses affects some of the paths.

**TABLE 6:** Effects for path model: males.

**Relationship**	**Model 1**				**Model 2**			
	**Effect and Sig**		**CI**		**Effect and Sig**		**CI**	
	**β**	***p***	**Lower**	**Upper**	**β**	***p***	**Lower**	**Upper**
PBC → Intention	0.518	^∗∗∗^	–	–	0.507	^∗∗∗^	–	–
Skill → Intention	0.245	0.006^∗∗^	–	–	0.228	^∗∗∗^	–	–
SubNorm → Intention	0.128	0.043^∗^	–	–	0.103	0.053	–	–
PBC → RiskProp → Intention	0.001	0.623	–0.010	0.024	0.001	0.650	–0.010	0.024
Skill → RiskProp → Intention	0.006	0.371	–0.010	0.061	0.006	0.348	–0.010	0.061
PBC → Motives → Intention	0.029	0.046^∗^	0.000	0.084	0.002	0.875	–0.029	0.041
Skill → Motives → Intention	0.033	0.056	–0.001	0.096	0.002	0.884	–0.044	0.038
SubNorm → Motives → Intention	–0.002	0.717	–0.036	0.022	0.000	0.684	–0.026	0.013
PBC → Motives → RiskProp	0.034	0.019^∗^	0.005	0.095	0.028	0.018^∗^	0.004	0.084
Skill → Motives → RiskProp	0.039	0.035^∗^	0.003	0.101	0.031	0.036^∗^	0.002	0.089
Motives → RiskProp → Intention	0.009	0.356	–0.016	0.058	0.009	0.285	–0.015	0.056
PBC → Motives → RiskProp → Intention	0.002	0.263	–0.002	0.013	0.002	0.187	–0.001	0.012
Skill → Motives → RiskProp → Intention	0.002	0.320	–0.003	0.016	0.002	0.262	–0.002	0.013

**β– Standardized estimates; ^∗∗∗^p = 0.001 or less; p is significant at the < 0.05 value.*^∗∗^*p* < 0.01; ^∗^*p* < 0.05. *Indirect effects: Bootstrapping: 2000 iterations and 0.95 bias–corrected; Direct effects: Maximum Likelihood Estimation.**

**TABLE 7:** Effects for path model: females.

**Relationship**	**Model 1**				**Model 2**			
	**Effect and Sig**		**CI**		**Effect and Sig**		**CI**	
	**β**	***p***	**Lower**	**Upper**	**β**	***p***	**Lower**	**Upper**
PBC → Intention	0.488	^∗∗∗^	–	–	0.466	^∗∗∗^	–	–
Skill → Intention	0.107	0.004^∗∗^	–	–	0.102	0.005^∗∗^	–	–
SubNorm → Intention	0.191	^∗∗∗^	–	–	0.184	^∗∗∗^	–	–
PBC → RiskProp → Intention	0.005	0.106	–0.001	0.020	0.005	0.109	– 0.001	0.020
Skill → RiskProp → Intention	0.015	0.025^∗^	0.002	0.035	0.015	0.025^∗^	0.002	0.035
PBC → Motives → Intention	0.013	0.077	–0.001	0.038	0.008	0.153	– 0.003	0.029
Skill → Motives → Intention	0.036	^∗∗∗^	0.016	0.072	0.027	0.004^∗∗^	0.010	0.058
SubNorm → Motives → Intention	0.016	0.013^∗^	0.003	0.039	0.008	0.077	–0.001	0.028
PBC → Motives → RiskProp	0.012	0.054	0.000	0.034	0.009	0.135	–0.003	0.029
Skill → Motives → RiskProp	0.033	^∗∗∗^	0.011	0.068	0.031	^∗∗∗^	0.011	0.065
Motives → RiskProp → Intention	0.019	0.024^∗^	0.003	0.049	0.019	0.025^∗^	0.003	0.049
PBC → Motives → RiskProp → Intention	0.001	0.050	0.000	0.004	0.001	0.102	0.000	0.004
Skill → Motives → RiskProp → Intention	0.003	0.018^∗^	0.001	0.010	0.003	0.019^∗^	0.001	0.009

**β– Standardized estimates; ^∗∗∗^p = 0.001 or less; p is significant at the < 0.05 value.*^∗∗^*p* < 0.01; ^∗^*p* < 0.05. *Indirect effects: Bootstrapping: 2000 iterations and 0.95 bias-corrected; Direct effects: Maximum Likelihood Estimation.**

For males, In Model 1, Motives mediates a significant, positive effect, between PBC and Intention, however, a more complex model shows that, at least in our context, it is not the case. In Model 2, none mediate any significant effect. That is, neither PBC, SubNorm, nor Skill make intentions for entrepreneurship significantly higher by personal motives or worth a risk when controlled for necessity; in fact, Motives becomes so low that the regression weights get close to zero on its mediation as well. We analyzed a path where we could measure whether PBC or Skill creates any effect on risk propensity through motives, which were significant and positive. This leads us to believe males may show higher propensity to take risk due to feeling competent and having motives, but this is not a significant drive for entrepreneurship, and may be just a reflection of their attitudes and a general drive for goals, as the paths that show not significant and with low coefficients are those to intention. Instead, it’s better explained by job security. We stress this is a contextual-specific effect, as it includes a highly relevant factor (necessity). Nevertheless, if the second model were to be ignored, the *p* value in the first is close to the significance threshold, which means that by itself the significance is not very clear. The confidence interval (CI) for this specific interaction is (0.000, 0.084), the lower bound suggests an extremely small effect, such that it is essentially the null value. Both models hint that Motives is not significant. To this, we rather suggest that male’s intrinsic motives for entrepreneurship may be strengthened by PBC, but JobSec seems to be better linked to intentions.

For females, perceiving themselves as skilled on entrepreneurship positive and significantly increases their motives for pursuing entrepreneurship, which further impacts intention. In other words, Motives mediates the positive effect of Skill. Perceived control does not by neither of the mediator variables. Motives also mediates a positive, significant effect between SubNorm and Intentions on Model 1, however, in Model 2 the effect becomes insignificant, which suggests that, under economic stress, peer opinion on entrepreneurship becomes less salient on developing motives, which could be our case.

RiskProp does not mediate a significant effect between PBC and Intention neither in males nor females, but it does between Skill and Intention in females. We ran a serial mediation path and found that skill positively affects (a) motives, which affects (b) risk taking propensity, and this in turn affects (c) intention in both models (In Model 1: β = 0.003, Model 2: β = 0.003). Skill is a determinant factor that makes females riskier and more desiring to pursue it (albeit by a small effect). We were expecting this to be true for both genders, which is not the case.

### Mean Comparison Between Males and Females

Lastly, Table 8 shows the mean for each of our variables by gender, as well results obtained from the t-test analysis for differences.

**TABLE 8:** Group means and *t*-test.

**Variable**	**Mean by groups**				***t*-test for Equality of Means**		
	**Group**	**Mean**	**Std. Deviation**	**Std. Error Mean**	**t**	***P***	**Mean Dif.**
PBC	Male	2.621	0.852	0.065	2.482	0.013	0.180
	Female	2.441	0.807	0.036			
Skill	Male	3.727	0.724	0.056	0.865	0.387	0.057
	Female	3.670	0.747	0.033			
SubNorm	Male	2.802	0.752	0.058	2.697	0.007	0.188
	Female	2.614	0.797	0.035			
Motives	Male	3.937	0.698	0.054	–3.324	0.001	–0.187
	Female	4.124	0.612	0.027			
RiskProp	Male	3.399	0.675	0.052	0.289	0.772	0.018
	Female	3.381	0.704	0.031			
Intention	Male	2.785	1.029	0.079	3.304	0.001	0.285
	Female	2.500	0.954	0.042			
Optimism	Male	3.922	0.592	0.045	1.508	0.132	0.089
	Female	3.833	0.688	0.030			
JobSec	Male	3.360	1.145	0.088	–0.805	0.421	–0.083
	Female	3.450	1.171	0.052			
LastRes	Male	2.570	1.263	0.097	1.735	0.083	0.192
	Female	2.380	1.243	0.055			

**Mean difference positive value means males score higher, negative value means females score higher. For t–test = Equality of variance is assumed in all variables: Levene’s Test = p > 0.05; 95% Confidence Interval for lower and upper values.**

The biggest difference in response comes from Intention, with a mean difference of 0.285 (significant, *p* < 0.001), and the smallest from RiskProp, with 0.018 (not significant, *p* = 0.772), Skills, with 0.057 (not significant, *p* = 0.387) was one of the lowest differences. We suspect this is due to the nature of the sample, as both have access to resources that allows them to hone skills commonly attributed, but not exclusive to entrepreneurs. Not surprising, both genders score low on Intentions, which is what the GEM 2019 report (Peña et al., 2019) found as well, and contrastingly, also on SubNorm. It also seems to follow the common trend of males scoring higher (and significant) on intentions, however, in our sample, females showed higher (significant) mean scores for Motives by 0.187. That is, they would pursue entrepreneurship to achieve certain intrinsic goals slightly stronger than males. Lastly, Table 9 resumes results obtained in contrast to our hypotheses.

**TABLE 9:** Hypotheses and results comparison.

	**Expectation**	**Result**	**Accept**
*H1*	PBC → Intention. Male = Female	**Confirmed**	O
*H2*	Skill → Intention. Male = Female	**Confirmed**	O
*H3*	PBC → Motives → Intention. Male > Female	**F*irst*** *model has significance issues in males, non-significant in females. Effect is* ***Not*** *significant on second model for neither*	X
*H4*	Skill → Motives → Intention. Male = Female	**No**. *Not significant in males*	X
*H5*	SubNorm → Intention. Male = Female	**Confirmed.** *Note: it is not significant in males on model 2*	O and X
*H6*	SubNorm → Motives → Intention. Female > Male	**No.** *Significant for females in first model*, ***not*** *in second.* ***Not*** *significant for males in neither*	X
*H7*	RiskProp → Intention. Male = Female	**No**. *Significant for females, not for male*s	X
*H8*	PBC → RiskProp → Intention. Male = Female	**No**. *Not significant for neither*	X
*H9*	Skill → RiskProp → Intention. Male = Female	**No.** *Significant for females, not for males*	X
*H10*	PBC → Motives → RiskProp → Intention. Male = Female	**No.** *Not significant in neither*	X
*H11*	Skill → Motives → RiskProp → Intention. Male = Female	**No.** *Significant for females, not for males*	X

**O, Hypotheses accepted; X, Hypotheses rejected. If both are present, it was confirmed in only one model, hence, partially supported.**

## Discussion

### Discussion of the Results

This study found that:

Perceived entrepreneurial skills between males and female university students are not significantly different from each other, and are moderate/adequate. These also statistically impact intentions.

As previously discussed, entrepreneurship skills are not exclusive for business students, and although entrepreneurship in education has either small or inconsistent effects (Martin et al., 2013; O’Connor, 2013; Bae et al., 2014) there is evidence that some things do have an effect, even for non-entrepreneurship-related or EE students, such as potential exposure to knowledge, behaviors and skills that are also useful for business (Langowitz and Minniti, 2007), and what has not been put into question is that a common trait between entrepreneurs is having high education (Bennett and Dann, 2000). Not-coincidentally, the highest percentage from students who express entrepreneurial intentions in Spain come from Social Sciences, not Business (Guerrero et al., 2016), hinting that knowledge or skills in itself are a valuable resource across many fields. Some other authors, like Hassan and Wafa (2012), have also found a diverse range of fields from where entrepreneurial intentions come from. Education also gives at the least, males and females alike, a window to develop competences, which could potentially eliminate hampering or discrimination due their lacking (Wilson et al., 2007). Also, the gender gap between perceived capabilities generally increases in innovation-driven economies (Kelley et al., 2014), however, the sample shows no significant difference in their mean, which could also hint higher education may work as a drive for indirect learning in some of these skills, and as an equalizer effect between genders.

As for causal effects, Bae et al. (2014) specifically found that entrepreneurial education itself does not have any more effect on intentions over females than males, however, as this study does not involve an entrepreneurship program (other than the closely related Business), we can only conclude that any byproduct of education that makes them feel competent creates a direct, but small, impact on intentions, not significantly different from each other.

Of course, the importance of this variable comes hand to hand with assumptions about the plasticity of entrepreneur formation, which must find a common ground between (1) studies that found biologically-linked traits that could potentially lead to entrepreneurial proclivities (Nicolaou et al., 2008; Zhang et al., 2009; Zhao et al., 2010), and (2) the presumption that people can be completely “scripted” from scratch to be entrepreneurs. In layman terms, having the competences by itself does not guarantee entrepreneurs; at best, it boosts their already-likely existing interest to act on it.

Males perceive others’ value for entrepreneurship (subjective norm) slightly higher, however, it predicts entrepreneurial intentions not significantly different between genders, and both score low. Motives for entrepreneurship mediates this effect to intentions on females, but not in males. This effect is not consistent when there are necessity-related responses controlled for. When they are, subjective norm is not mediated by motives on either, and its direct effect is not significant in males, suggesting external valuation on entrepreneurship may less relevant under economic stress to determine intentions, and may be a potential explanation for its influence (or lack thereof) on intentions in both genders.

How social circles view entrepreneurship affects intentions slightly (and not substantially) different between males and females, according to a meta-analysis realized by Haus et al. (2013) using developed countries. More specifically, the results from the first model in this study may reflect those closest to Baughn et al. (2006), which it is more influential over females (who also enjoy of relatively high gender equality), as it increases their motives. However, as environmental variables come into the equation (i.e., uncertainty and unemployment): these motives have a non-significant mediation effect. The impact of subjective norm has been previously questioned (e.g., Krueger et al., 2000; Autio et al., 2001; Turker and Sonmez Selcuk, 2009), and these results reasonably position its effect as circumstantial: people would find less value in it under stress of unemployment.

There are not currently many studies on the effects of environmental stress due to necessity on intention toward entrepreneurship, however, Arrighetti et al. (2016) obtained similar results in an Italian sample. Specifically, that family are not an essential component, but instead their support stems from educational intuitions. We argue, because of this, that how they perceive support from all types of institutions (government, private, and educational) would be important, and perhaps a better focus than the support from people in some cases. If this holds true, the implication for institutional (mostly governmental and financial) support for entrepreneurship in Spain is a serious issue, which should be equally as positive as the educational support students are currently perceiving. It is currently not (Guerrero et al., 2016).

PBC predicts significantly stronger than entrepreneurial skills and subjective norm on entrepreneurial intentions in both genders. None were significantly different between genders. Nevertheless, how this variable plays with other perceptions is dynamically different between males and females.

This variable shares high commonality with self-efficacy (Ajzen, 2002) which tends to differ between gender; at least the degree how they perceive it (Chen et al., 1998), as well in PBC (Maes et al., 2014), so this was not unexpected. Although the mean difference obtained is statistically significant between males and females, it’s not particularly high in neither. Also, given educational resources and skills are present in the sample (yet yields low values for both), we cannot view its relationship as positive as some authors, like Zhao et al. (2005), have found, where self-efficacy was significantly impacted by education (although this is likely because the variable used here was a composite predominantly oriented toward controllability, which could also be impacted by other factors such as finance or institutional support), which is what Guerrero et al. (2016) found in the Spanish context.

Other theoretically possible explanations for gender differences and low values in entrepreneurial PBC could be: perceptions due to personality and/or temperament (Lippa, 2010; Zhao et al., 2010), lack of experience (Miralles et al., 2015), limited exposure to role models and other entrepreneurs (BarNir et al., 2011), or low social support (that also scored low as Subjective Norm), such as lack of regional support or high bureaucracy, which is present in Spain (Guerrero et al., 2016), and/or a perceived gender barrier.

From this study though, we can only conclude that even under an educational context, both score low (and females lower), and perceiving competences by itself does not seem to be enough to put their PBC or efficacy in a positive light.

As for causal paths, the likelihood of males and females developing intentions due to PBC is actually not significantly different from each other, which suggests this variable is equally important for both in order to develop some determination toward being self-employed. This result was also found by Zhao et al. (2005)

A gender difference, however, does seem to surface is how impactful it is to other variables that also influence intentions. This is also the case for perceived skills, which we discuss in the following:

Females who perceive themselves as entrepreneurially-skilled have higher intentions because it also strengthens their motives to pursue entrepreneurship, and are willing to be more risky toward achieving them. This is not the case by PBC, suggesting their push toward certain entrepreneurial beliefs may be more resource-dependent than belief-based. This effect is consistent whether there are or not necessity-related responses controlled for, suggesting necessity, risk taking and an intrinsic motives toward entrepreneurship may be intertwined. A serial mediation effect from motives and risk propensity between skill and intention is significant, whether there are necessity-related responses controlled or not.

Males PBC of entrepreneurial endeavors does not increase intentions because is strengthens their motives, nor perceiving themselves as entrepreneurially-skilled. Neither of these variables increases their intentions as an effect of becoming riskier either. This effect is not consistent when there are necessity-related responses controlled for. When they are, neither their motives, nor their willingness to take risk, become impactful in any way whatsoever toward entrepreneurial intentions, nor mediate any effect, suggesting they are better driven in our sample by job security.

Females answered in a way that, the more skillful they feel, the higher they would perceive their propensity to take risks, which also significantly impacts their intentions for business. This same statement applies for their motives to pursue entrepreneurship. This means both beliefs get pushed by perceiving highly their abilities. Langowitz and Minniti (2007) also found a relation between skill and intention in female students, and others, like Kickul et al. (2008) did with high school students. We further strengthen and boost on these findings by including these indirect effects. Unexpectedly, this is not what we found for males, neither by skills or PBC. It is worth noting that their answers for risk taking propensity are not significantly different from females, which means is not because they wouldn’t be risk takers themselves.

Risk taking propensity as an entrepreneur trait has been previously questioned (Brockhaus, 1980), and have found its usefulness as phase-specific (i.e., only for intentions; Zhao et al., 2010). These results indicate some significant relationship (and only in one group), although, like some of them would argue, it does not hint as an entrepreneur trait neither. Risk taking behavior varies according to its context, regardless if people rate themselves as high risk takers (Nicholson et al., 2005).

It’s not very likely that this case points to excitement for novelty or sensation-seeking for riskiness, as it’s sometimes attributed to entrepreneurs [which has also been linked to biological responses related to certain risk-taking behaviors, but usually involving danger (Zald et al., 2008), which we seriously question it’s the entrepreneur case]. In fact, people in Spain rank higher than their European neighbors in fear of failure (Peña et al., 2019), and has been expressed as a reason for not being entrepreneurs, more so by females (Sánchez Cañizares and Fuentes García, 2010; Alemany et al., 2011; Peris-Ortiz et al., 2014). This hints the relation to riskiness in this case may be out of a different reason.

The relation would likely sum up to whether people dare to stand up and take risks as a composite of, both, an adaptive response to high uncertainty in the country, such as that given by unemployment, and a personal likeability for business. Unlike males, necessity responses do not dampen any path on females. What was found with the female sample is that the effect of skill, combined with an increase of personal motives and risk propensity to intentions is present regardless of the effects of necessity, suggesting they generally become riskier toward venture creation to avoid unemployment, while finding achievable personal goals through it (which also gets impacted by the prospects of job security). In other words, because they want it and because they could use it to evade uncertainty. For the male sample, this does not seem to be the case, and works dichotomously: being driven by necessity factors, but not for personal goals or motives. Instead, it looks as if males may be considering entrepreneurship as a hypothetical second option or failsafe, which may explain the non-significant relationship of risk-taking with intentions.

Results also show females have stronger motives for entrepreneurship than males, which could be due to some reasons. The first instinctual explanation would be because it’s an artifact defect, as the variable lacks some items that have been found of importance to males, such as economic ambition or the inherent challenge of what implies creating a business (Maes et al., 2014). In other words, the variable slightly favors females. While this may be valid argument, however, it is also linear thinking to fit results to an assumption: that males are always supposed to find it more attractive, which can be a defect in reasoning. The item composition of this variable shows females actually responded higher in all four of its items, and the difference in their mean is significantly different. It can be said for certain the female sample is looking for independence, novelty, and a feel of personal achievement as goals through entrepreneurship slightly higher than males, and these impact their intentions, but not in males.

We believe this is partly due to field demographics. Males and females distribute among different business sectors (Klapper and Parker, 2011), the latter more prone to create small, single handed business (Coleman, 2007), which is the most common type in Spain (Peña et al., 2019). Second, more than half of the female participants are education or social-related (i.e., Psychology) students, which is a female-dominated market in business (Kelley et al., 2017). These results are logically reasoned if they their motives to pursue entrepreneurship are higher, and means there are likely some sectors where females are looking for entrepreneurship because it fills them as individuals more than in males. Irrelevant of the size of the difference, this is important for female entrepreneurship literature.

### Theoretical Contributions to the Entrepreneurship Literature

The contributions of this study to entrepreneurship literature are at least twofold. First, it contributes the latest report on student’s beliefs toward entrepreneurship in Spain (Guerrero et al., 2016) by creating causal paths that show how some of their current beliefs relate to Intention. We went beyond this and analyzed them by each gender separately.

We also show there’s certain attitudinal variability by gender, finding some of the influence of beliefs to intention in females are pushed by skills, but in males by confidence, more so, their personal motives for entrepreneurship also vary by these beliefs. It is also the case for risk taking propensity in females, although not in males by neither competence nor confidence. This suggests a certain division of competence/confidence relation between genders that should be further explored.

Second, we expand in how necessity acts in entrepreneurial intentions and how it varies by gender. We found that in both, males and females, part of their determination toward business is caused by the need of guaranteeing themselves a job, but contrary to what it is generally expected, its females who seem to be interested in it for their personal growth and gains. This paradigm shifter could be because artifact issues, but it also hints that it’s not always the case that entrepreneurship is something males want, and that women do out of necessity. In fact, this study showed the opposite: it is males the ones who are looking for it by necessity-related reasons, possibly as a second-choice to employment. We believe intention-related research should focus on female-dominated demographics in academic contexts, and move away from the general studies done with business students, as some results change, and our results also hint entrepreneurial competence is not focused exclusively on business programs.

We also contribute to the pool of studies that show subjective norm is not impactful toward intention, however, I add that this seems partially related to how necessity-related motives play in high-unemployment environments.

The worst case notion of looking for entrepreneurship due to all possible employment opportunities being exhausted is not the case for students in Spain, and it predicts negatively on intentions, although only significantly in males.

### Practical Implications

This study shows that Spanish students are perceiving entrepreneurship as means of job stability, and some of their intentions is explained by this, however, informal institutions like the social perception of entrepreneurs, as well formal institutions, such as government support, are not favoring these intentions. Policy-implications are well exhausted in literature, and there definitively is already an approach to dampen many institutional-related issues in Spain, as discussed in the theoretical review. Recommendations from this side can be simplified as: evaluate EE programs and further push the cross-sectional/transversal approach to visualize entrepreneurship as a feasible career and further expose students to the idea, strengthen the visibility business accelerators and orientation centers for future business and smooth the business registration processes, as these could start to give a better impression that entrepreneurship is being supported.

Specifically, government institutions should be making visible even stronger the idea of small business, as they likelihood of they pursuing these type of venture under these scenarios becomes higher (Hofstede et al., 2004), and would dampen the type of entrepreneurship of self-employment. Females are also not just valuing their skills, but also looking into the intrinsic prospects of entrepreneurship. Small business, investors and female entrepreneurs have shown to have a hard history (Klapper and Parker, 2011), due to the naturally lower gains obtained from small ventures, as well some bias for female traits (Balachandra et al., 2017). We can’t say from this study how these are relevant for creating intentions, but it generally proves to be a challenge at the nascent stage.

While a mind state to ensure small business and entrepreneurs be on the positive side of financial investors, as well governmental policies to support firm entry, most of these policies are already present in Spain, and the suggestions should go beyond the typical ideas. Therefore, our definitive suggestion focuses on practicality: to tap into the virtual side of entrepreneurship and exploit more recent approaches for finance and business promotion, as it has proven to be an effective mean to pursuit not only novel ideas, but to obtain resources for small projects (Mollick, 2014). Spain is innovation-driven, but the number of innovative businesses are actually low (Peña et al., 2019). Spain should behave innovation-driven.

Given the student nature of our population, we strongly suggest to evolve at the educational institutional level: further promote and expose crowd-source concepts, like Crowdfunding, at universities. These are forms of venture that are funded by the crowd, or users, in a platform where money is pleaded, and accordingly pledged by this crowd in accordance to their interests. The control for the project is, in most of its sense, at the hands of its creator and the community it wishes to aim (Mollick, 2014). Due to the virtual nature and the highlight of pitching by the backers, or crowd, we recommend to exploit the opportunity to teach and train students into successfully pitching their projects could potentially make virtual entrepreneurship a brewing spot for finance and innovative projects. These virtual spaces have less restriction of ideas, and more probability to find support niches. If students are looking to entrepreneurship as means to fight off economic issued in Spain, every viable option should be considered. We strongly suggest to move into modern approaches, where communities more akin to each individual support their business ideas, as well be able to receive direct support and feedback from their potential funders, whom may also be better group-representatives (best exemplified by Pinkstarter for minority groups). Other virtual alternative platforms, such as Patreon, have also shown fruitful for support and startups.

In fact, some of these, like Crowdfunding has shown to not have a negative female bias (Josefy et al., 2016), and one study even found it favors female communication style more than males’ (Gorbati and Nelson, 2015), as well removes geographical barriers (Agrawal et al., 2011), meaning there is a higher chance of students to pursue their business ideas without relying solely by the low support from political institutions and the current social norm in Spain. While policies are important (and some are specifically aimed to support these type of crowd-sourced concepts), we believe a strong contender to increase entrepreneurial potential in Spanish university students is to give them more individual control of the entrepreneurial process, where they could feel there’s something they can do for their creative ideas. This is what an innovation-driven society should be aiming at.

### Limitations and Suggestions for Future Research

Our study maps relations between some perceptual variables and how they relate between gender in a Spanish (or perhaps a similar) context. Although some of our findings may prove useful to understand how certain environments affect intentions differently on each gender, we do not believe this is a complete picture of its dynamics, and believe some other variables that were out of our reach should be used to further explain relationships. We suggest to further complex the model by: exploring the drive of intentions on males and females by including beliefs on entrepreneurship (Krueger, 2017), creativity and innovation, opportunity recognition, caveats such as difficulties or barriers due to bureaucracy impact on intentions. Theories, such as Precluded Interest Theory and Expectancy Theory could be explored, as well contrasted, given they intend to explore how people “fit” into occupations because of how and who they are. Additionally, our sample was not proportional in the number of male and female participants, meaning a bias in sample proportion may account for some of the results. To strengthen these results, further studies could look to have a more equitable sample between the two.

Due to artifact limitations, we could not distinguish whether our sample framed their answers by thinking of entrepreneurship or self-employment, which have a clear distinction (Acs, 2006). Hofstede et al. (2004) found that unfavorable environments for entrepreneurship generally creates dissatisfied individuals who look for self-employment for realization, not entrepreneurship, and generally leads to small business. GEM’s 2019 report shows most enterprises in Spain actually fit this model, TEA being dominated by small business, predominantly single-handed, followed by the less than five employees type. This hints our study was likely answered by framing a self-employment reference, however, this is speculative. A further effort needs to be done to distinguish how people answer between the two.

We controlled for the effects of necessity due to having at our availability certain items that measure responses related to it, however, these are two single items, which we could not test for reliability. Although they are considerably unidimensional and can be used (Bergkvist and Rossiter, 2007; Bergkvist, 2014), a further effort to have multiple-item constructs related to both factors would allow to calculate reliability values for each.

## Data Availability Statement

The raw data supporting the conclusions of this manuscript will be made available by the authors, without undue reservation, to any qualified researcher.

## Ethics Statement

Ethical review and approval was not required for the study on human participants in accordance with the local legislation and institutional requirements. The participants provided their written informed consent to participate in this study.

## Author Contributions

AW: writing, statistics, and discussion. BH-S: sampling, writing, and discussion. JS-G: statistics, sampling, and discussion.

## Conflict of Interest

The authors declare that the research was conducted in the absence of any commercial or financial relationships that could be construed as a potential conflict of interest.
